# Analysis of volatile compounds by GCMS reveals their rice cultivars

**DOI:** 10.1038/s41598-023-34797-2

**Published:** 2023-05-17

**Authors:** Shengying Hu, Hongbo Ren, Yong Song, Feng Liu, Lili Qian, Feng Zuo, Li Meng

**Affiliations:** 1 Engineering Research Center of Agricultural Microbiology Technology, Ministry of Education, Heilongjiang University, Harbin, 150500 China; 2 Key Laboratory of Molecular Biology of Heilongjiang Province, College of Life Science, Heilongjiang University, Harbin, 150080 China; 3 Quality and Safety Institute of Agricultural Products, Heilongjiang Academy of Agricultural Sciences, Harbin, 150086 China; 4 Shandong Yanggu Huetai Chemical Co., Ltd., Shandong, 252300 China; 5 College of Food Science and Technology, Heilongjiang Bayi Agricultural University, Daqing, 163319 China

**Keywords:** Metabolomics, Chemical biology, Biogeochemistry

## Abstract

Due to the similarity in the grain and difference in the market value among many rice varieties, deliberate mislabeling and adulteration has become a serious problem. To check the authenticity, we aimed to discriminate rice varieties based on their volatile organic compounds (VOCs) composition by headspace solid phase microextraction (HS-SPME) coupled with gas chromatography mass spectrometry (GC–MS). The VOC profiles of Wuyoudao 4 from nine sites in Wuchang were compared to 11 rice cultivar from other regions. Multivariate analysis and unsupervised clustering showed an unambiguous distinction between Wuchang rice and non-Wuchang rice. Partial least squares discriminant analysis (PLS-DA) demonstrated a goodness of fit of 0.90 and a goodness of prediction of 0.85. The discriminating ability of volatile compounds is also supported by Random forest analysis. Our data revealed eight biomarkers including 2-acetyl-1-pyrroline (2-AP) that can be used for variation identification. Taken together, the current method can readily distinguish Wuchang rice from other varieties which it holds great potential in checking the authenticity of rice.

## Introduction

Rice (*Oryza sativa* L) is the staple food for approximately 3.5 billion people worldwide with an estimated production of 480 million tons annually^1^. Rice provides up to 50% of the dietarycaloric supply and a substantial part of the protein intake for about 520 million people living in poverty in Asia^2^. The market value of rice is primarily determined by the grain quality, which in turn depends on the genetic background, agronomic management, geographic factors and postharvest factors^3,4^. Although the grain quality and thus the price for rice of different geographic origins could differ vastly, their appearance may be very alike. Thus, deliberate mislabeling and adulteration represent a serious issue, which not only threatens the credit of the traders, but also infringes the right of consumers^5^. To prevent such actions, a high-throughput, sensitive and precise method is urgently needed to discriminate rice of different geographic origins. The key to determining the geographic origin was to identify specific biomarkers for a specific rice variety. Previous studies had used a wide range of properties such as stable isotope, mineral element content that are associated with geographic features to track the origin of rice^6,7^. Notably, multiple parameters can be used together as they may provide complementary information. For instance, the isotope composition of δ^13^C, δ^15^N, δ^2^H, δ^18^O had been employed in junction with the multi-elemental concentrations analysis to determine the origin of different rice varieties^8^. For a more comprehensive comparison of the components among various rice cultivars, spectroscopy approaches such as near-infrared reflectance spectroscopy (NIR), nuclear magnetic resonance spectroscopy (NMR), Raman spectroscopy (RS) had also been used^9–11^. These methods requires large amount of input for data collection and has no direct relationship with the quality of rice.

Volatile organic compounds (VOCs) are the main source of aroma in rice. Although difficult to describe by words, aroma differ greatly among varieties due to a different composition in VOCs. Indeed, a previous study has reported the geographical discrimination of rice based on VOCs^12^. Headspace solid phase microextraction (HS-SPME)was applied in volatile substance concentrate to improve the analytes placed on column^13^. Due to its simplicity, high sensitivity and reproducibility, SPME is particularly suitable for the analysis of volatiles in food^14,15^. Coupled with GC–MS, the aroma compounds can be identified precisely.

Different rice varieties have different aromatic characteristics and volatiles of rice can be considered to be biomarker candidates of geographical varieties determination. Rice considered to be has high quality because of the attractive aroma and good taste^16^. So, to characterize the aroma profile of fragrant rice is meaningful not only on adulteration but also good for breeding. Wuchang rice, as its name suggests, grows in Wuchang county in Heilongjiang province of northeast China^17^. It is known for its intense aroma and enlisted as a National Geographical Indication Product of China^18^. Wuyoudao 4 is the dominant variety in this area since 1990s. Despite big differences in composition and the grain quality between WuChang rice and other varieties, it is still challenging to distinguish them due to a lack of robust and sensitive method^19–21^.

In this study, we aimed to address this challenge by profiling Wuchang rice VOCs with HS-SPME extraction and GC–MS detection. First, an untargeted metabolomic approach was used to profile the VOCs in both WuChang and non-WuChang rice varieties. Subsequent multivariate analysis showed an unambiguous classification of the samples according to their geographic origins. In addition, our data led to the identification of eight VOCs that can be used as biomarkers in variety determination. The GCMS approach described is readily applicable to determine the geographic origin of other premium rice.

## Materials and methods

### Material

Twenty rice samples grown in 2018 were purchased from either Wuchang or other regions (see Table 1 for details). It was confirmed that all methods were performed in accordance with the relevant legislation. For each sample group, three biological replicates were obtained. All samples were stored at – 80 °C until analysis. Headspace vials (20 ml in volume) were purchased from Agilent, each with a silver aluminum cap and a polytetrafluoroethylene (PTFE)/silicone rubber septum. Supplies for solid phase microextraction (SPME) including a manual injection handle and a 75 μm carboxen/polydimethylsiloxane (CAR/PDMS) extraction head were purchased from SUPELCO (St. Louis, USA)^22^.

**Table 1:** The origins of the 20 rice samples.

Sample	Origin	Number	Cultivar
Wuchang rice	Changbao	CB	Wuyoudao 4
	Yingchengzi	YCZ	Wuyoudao 4
	Longfengshan	LFS	Wuyoudao 4
	Lalin	LL	Wuyoudao 4
	Minle	ML1	Wuyoudao 4
	Minle	ML2	Wuyoudao 4
	Anjia	AJ1	Wuyoudao 4
	Anjia	AJ2	Wuyoudao 4
	Xiangyang	XY	Wuyoudao 4
Non-wuchang rice	Jiansanjiang	JSJ	Kongyu 131
	Zhaoyuan	ZY	Songjing 22
	Xiangshui	XS	Shangyu 397
	Jilin	JL	Jijing 83
	Panjin	PJ	Liaojing 9
	Sheyang	SY	Huaidao 5
	Hubei	HB	Liangyoupeijiu
	Jiangxi	JX	Jinyou 463
	Henan	HN	Yuandao 109
	Korea	HG	Dongjin
	Thailand	TG	Jasmine 105

### Extraction of volatiles by HS-SPME

Four grams of rice grain were stored in a 20 mL headspace vial. The headspace bottle was heated at 80 °C for 30 min in a water bath. Subsequently, a SPME fiber was inserted into the headspace portion of the vial and then incubated for 60 min to absorb VOCs from the rice grain^23^.

### GC–MS analysis

GC–MS analysis was performed using a TRACE GC ULTRA (Thermo) coupled with a SQ QUANTUM XLS (Thermo). The SPME fiber was inserted into the inlet of the GC–MS with the fiber head pushed out, and VOCs were desorbed at 250 °C for 5 min. Helium was used as the carrier gas with a flow rate of 1 mL/min and a spitless injection was used. A DB-5 capillary column (30 m × 0.25 mm × 0.25 μm) was used for separation with the following temperature gradient: an initial temperature of 40 °C for 3 min; a linear increase to 100 °C at 5 °C/min and hold for 3 min; and ramping to 250 °C at 10 °C/min and hold for 4 min. Eluted compounds are ionized by electron ionization with an electron energy of 70 eV and an ion source temperature of 230 °C. Full MS scans were performed at the mass range of 40–300 m*/z*^24^*.*

### Chromatographic analyses

The obtained MS spectra were matched to the National Institute of Standards and Technology reference spectra (NIST 08 and Wiley 7). The retention index (RI) of putative compounds is further compared to previous reports (Table S2). The normalized peak area was used for calculating the compound concentration. Data from three biological replicates were reported (Table S3).

### Clustering analysis

Prior to statistical analysis, the raw MS intensities were first subjected to log transformation and Pareto scaling. Feature selection was performed using standard methods such as fold change (FC), volcano plot, t-test and empirical Bayesian analysis of microarray (EBAM). Features with a relative standard deviations (RSD) > 30% were removed from further analysis. A feature was considered to be differentially expressed with the following criteria: (1) FC < 0.8 or FC > 1.2; (2) p-value < 0.05) and (3) false discovery rate (FDR) < 0.1.

Euclidean distance-based hierarchical cluster analysis (HCA) was used to explore the variance distribution and classification patterns among samples with the Ward method^25^. Discriminant analysis of the rice samples was performed with principal component analysis (PCA), partial least square-differential analysis (PLS-DA), cross validation and Heatmap. Further evaluation of the accuracy of these predictions were performed by random forest analysis^26^. Finally, putative biomarker candidates to discriminate Wuchang rice from other varieties were chosen based on (1) a variable importance in the projection (VIP) score ≥ 0.8; and (2) differentially expressed in the univariate analysis^27^. All the data were analyzed were conducted by R 4.2.2.

## Results and discussion

### The shape and VOCs profile of the rice grain

**Figure 1:**
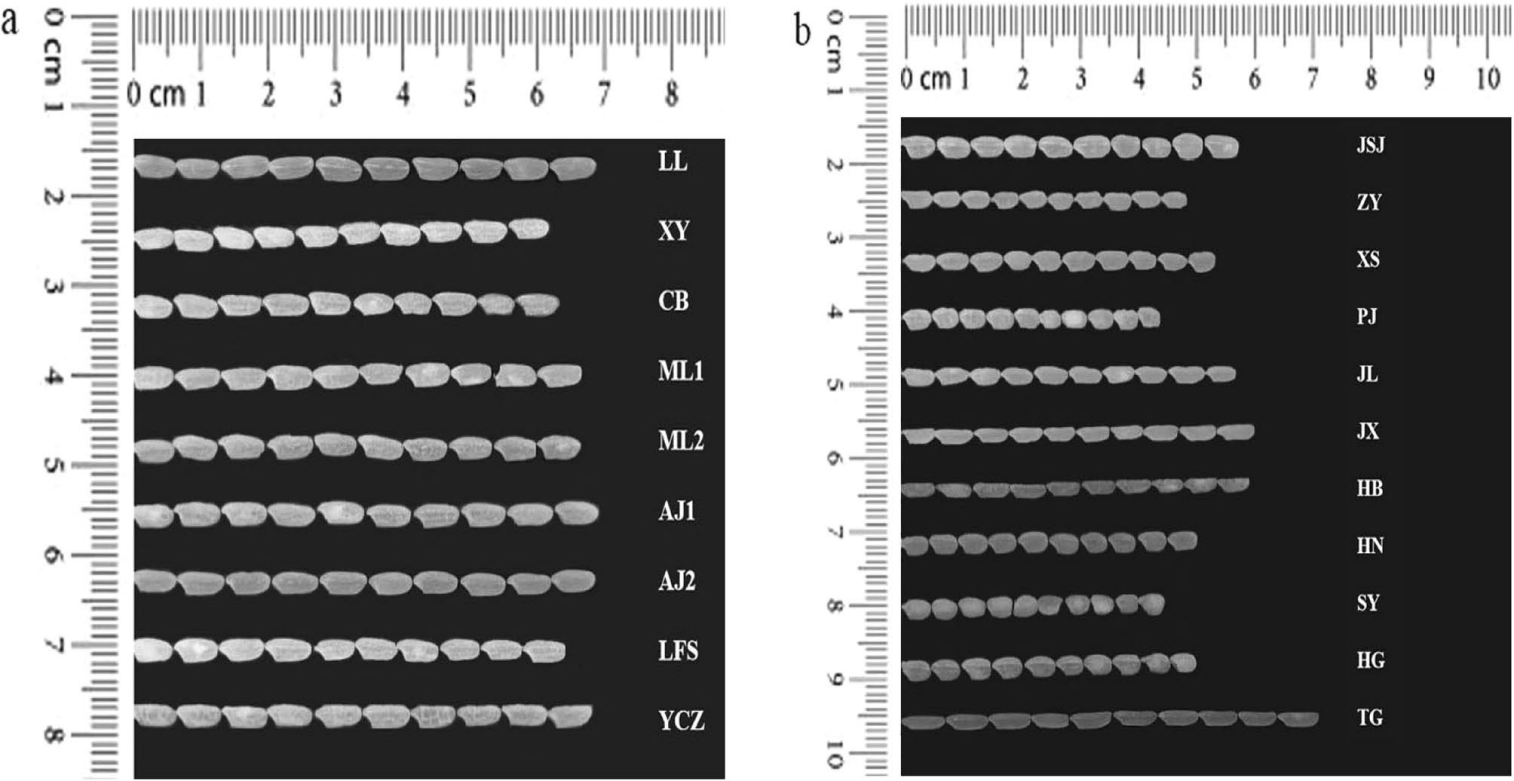
The morphological characteristics of difference rice grains. (**a**) The wuyoudao4 growed in 11 sites of Wuchang (**b**) The cultivars from Non-wuchang origins.

**Figure 2:**
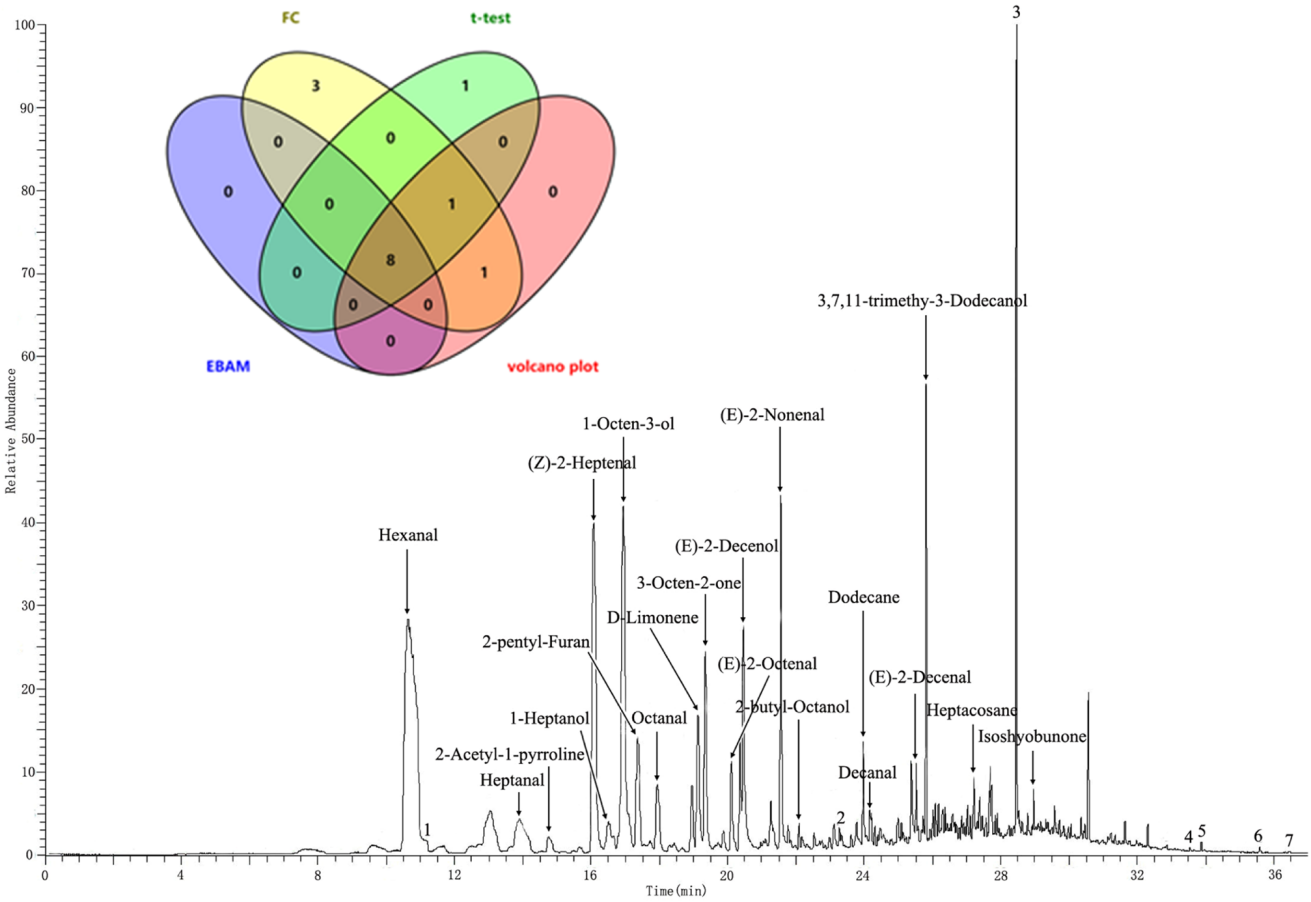
VOCs profiling of Wu Chang rice obtained by GC–MS using CAR/PDMS SPME.

The morphology of rice showed in Fig. 1. Some of the rice have different shapes with Wuyoudao4, but some cultivar has similar length and shape such as JSJ. Although Wuyoudao4 have the characteristics with long grains, but it is also hard to tell whether it is Wuyoudao4 only depend on the shapes by naked eye. To prevent mislabeling and adulteration, we developed a method to profile VOCs from Wuchang rice and non-Wuchang rice. The representative total ion chromatography was shown Fig. 2 with the peaks annotated. In total, 22 VOCs were identified from our samples. These VOCs covered compounds with a diverse range of chemical compositions including eight aldehydes, five alcohols, two ketones, two heterocyclic compounds, and five hydrocarbon compounds (Table 2). In addition to VOCs, siloxane derivatives were also observed due to the presence of PDMS in the SPME fibers (Table S2), which is consistency with the previous report^28,29^.

**Table 2:** 8 typical biomarkers characteristics of Wuchang rice.

Retention time (min)	Compound name	Formula	NIST match (%)	Retention index (RI)^a^		RSD (%)	VIP Score	T-test		FC
				Experiment	Reference			P-value	FDR	
14.06	Heptanal	C_7_H_14_O	93	839	844	28.93	2.33	0.032	0.035	2.33
14.76	Acetyl-1-pyrroline	C_6_H_9_NO	96	897	901	5.22	3.41	0.0002	0.004	8.69
16.53	1-Heptanol	C_7_H_16_O	92	739	754	27.97	0.99	0.028	0.017	0.76
17.98	Octanal	C_8_H_16_O	93	907	941	5.81	0.89	0.043	0.037	1.25
19.35	3-Octen-2-one	C_8_H_14_O	92	800	810	27.96	0.98	0.049	0.034	0.59
20.38	(E)-2-Decen-1-ol	C_10_H_20_O	90	778	790	10.36	1.34	0.016	0.013	1.47
25.42	(E)-2-Decenal	C_10_H_18_O	91	804	856	28.35	0.88	0.028	0.024	1.24
25.82	3-Dodecanol,3,7,11-trimethyl-	C_15_H_32_O	90	780	817	19.15	0.89	0.036	0.035	1.22

^a^RI indicates retention index. Reference RI values were obtained from https://www.nist.gov/.

### Chemometric analysis of biomarkers for rice screening

To identify putative biomarker in determining the geographic origin of rice, we performed both single factor and multivariate analyses on the VOC dataset. First, differential analysis revealed that the concentration of eight features were significantly different among samples (p < 0.05, FDR < 0.1, and FC < 0.8 or FC > 1.2) showed in Venn diagram (Fig. 2). This indicates that the profile of VOCs could be used to distinct rice varieties.

**Figure 3:**
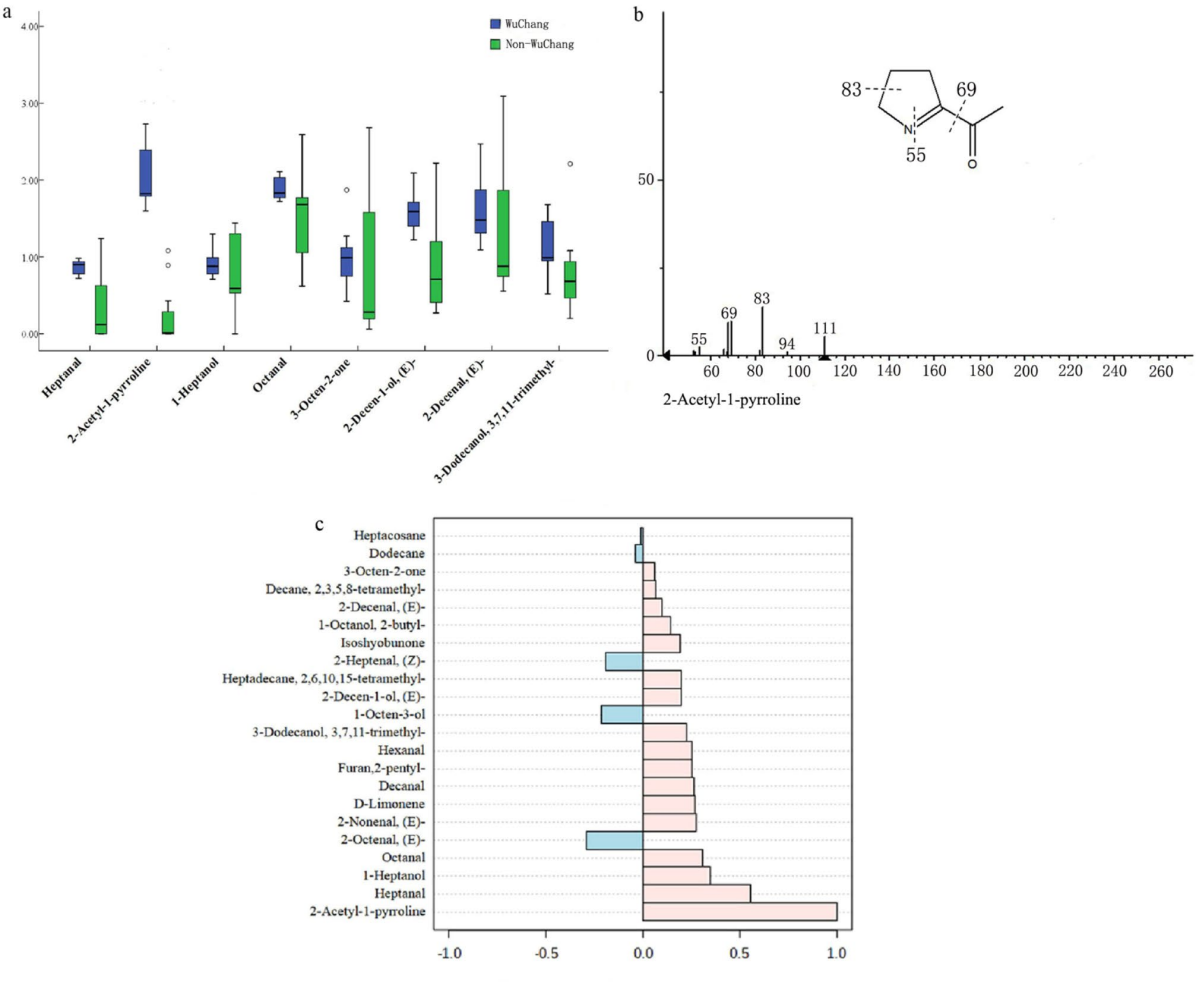
Box plots and 2-AP analysis of eight chemical biomarkers in rice samples. (**a**) Box diagram of eight typical biomarkers for rice samples from WuChang and other regions; (**b**) 2-AP ion map; (**c**) Correlation analysis of 2-AP with other volatile compounds.

The eight features showing differential concentration between Wuchang and non-Wuchang rice were further evaluated by the variable importance in the projection (VIP) score. All of them passed the 0.8 cutoff and were thus considered as putative biomarkers. They consisted of three aldehydes (heptanal, octanal, (E)-2-decenal), 3 alcohols (1-heptanol, (E)-2-decen-1-ol, 3,7,11-Trimethyl-3-dodecanol), one ketone (3-octene-2-one), and one heterocyclic compound (2-acetyl-1-pyrroline, or 2-AP). Details of the these compounds were summarized in Table S1. Compared to non-Wuchang rice, Wuchang rice showed a significantly lower level of 1-heptanol and 3-octene-2-one (P < 0.05), and a much higher level for the remaining six putative biomarkers (Fig. 3a). Among them, 2-AP has been reported as an important chemical trait to differentiate fragrant rice from non-fragrant rice. Other non-wuchang rice have a relative lower amount of 2-AP amount except TG(Jasmine 105). The identification of 2-AP (Fig. 3b) wast validate which the robustness of our method, but also aligns aligned well with previous findings.

Despite we observed a significant difference in the 2-AP concentration between Wuchang and non-Wuchang rice, we next sought to see whether other volatiles show a similar pattern. Thus, we examined the correlations between 2-AP and other compounds. We observed a negative correlation for (E)-2-octenal, (Z)-heptenal, 1-octene-3-ol, dodecane, and heptadecane, and a positive correlation for the rest of 22 VOCs (Fig. 3c).

### Evaluation of biomarker efficiency

Previous studies reported the use of 2-AP in determining fragrant rice and non-fragrant rice^30^. However, the usefulness of other biomarkers remain unclear. To this end, we assessed the discrimination efficiency of putative markers identified here using several multivariate analysis techniques. Our results showed that a clear separation of rice samples by the VOC data. Finally, random forest (RF) analysis further revealed a high accuracy of these markers in distinguishing rice samples from different sources.

### Clustering and heat map analysis

**Figure 4:**
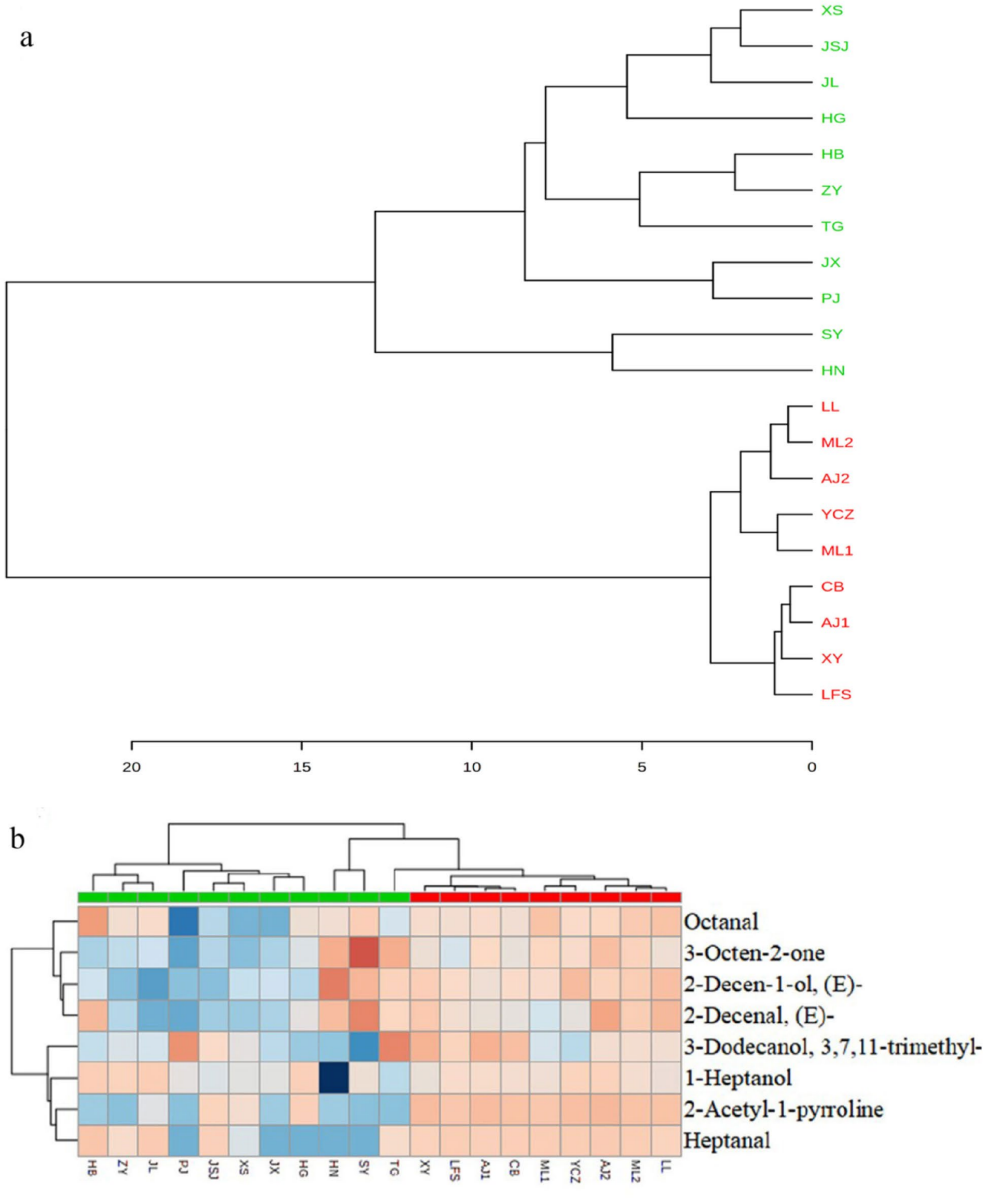
Clusting analysis of rice from different regions using eight VOC typical biomarkers. (**a**) Dendrogram (**b**) heatmap, weak blue and white represent weak correlation, pink and brown represent strong correlation.

HCA clustering classified the samples into two groups neatly with respective to their geographic origins: Wuchang rice and non-Wuchang (Fig. 4a). A large distance between the two groups was also observed (Euclidean distance > 20), supporting the significant difference in grain quality between them. By contrast, the Euclidean distances among the 8 Wuchang samples are less than 5, suggesting that subtle variations exist for rice grown in the same area.

Next, heatmap analysis was performed to visualize the correlation of eight putative VOCs (Fig. 4b). The 20 samples were separated into two groups. A high degree of correlation was found for the first group consisted of 9 samples, including all 8 varieties from Wuchang. Notably, samples in this group were also high in 2-AP, contribution to a better rice flavor. By contrast, a lower level of correlation in term of VOC content was found for the second group of 11 samples.

**Figure 5:**
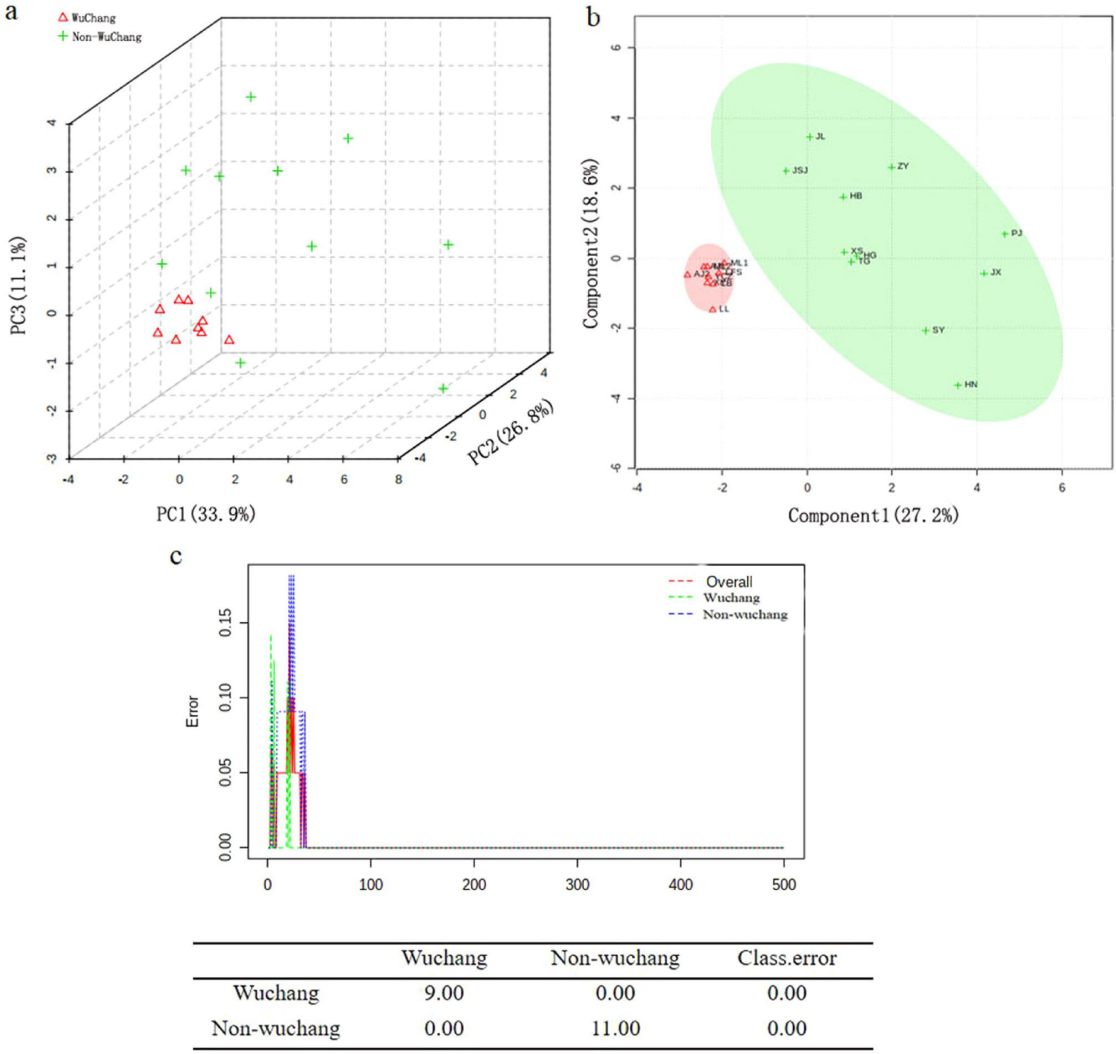
Different statistical analyses of the chemical biomarkers. (**a**) 3D score plot between the selected PCs in PCA. (**b**) PLS-DA models (**c**) Cumulative error rates by Random Forest classification.

Unsupervised PCA was used to process the original data into orthogonal components. The results showed a percentage of variance of 33.9%, 26.8% and 11.1% for the first three PCs respectively (Fig. 5a). Consistent with the clustering analysis, a clear separation of Wuchang rice from other varieties was also observed using the first three PCs. It is notable that nine samples of Wuchang rice concentrated in the three-dimensional PCA plot, supporting that no statistical significant difference were found among them (p > 0.05). Conversely, a wide distribution was observed for the 11 other rice samples, reflecting the fact that they were grown in a diverse range of geographic locations.

Similar to clustering and PCA, the PLS-DA analysis showed a clear aggregation among Wuchang varieties (Fig. 5b), indicating that the composition and content of these nine samples were similar. By contrast, disperse of the other 11 samples suggested a large difference in composition and content among them. Significantly, the PLS-DA also revealed the discrimination capacity of the eight biomarkers with a fitness R^2^ of 0.90, prediction goodness Q^2^ of 0.85, and displacement test p value of 0.01.

To further evaluate the ability of the eight putative biomarkers in rice classification, we built a model based on the random forest algorithm (RF). RF is not only suitable for feature selection, but also provides useful information such as OOB (out-of-bag) error, variable importance measurement, and outlier measurement^31^. We found an excellent classification ability of the eight putative biomarkers, indicated by a classification error of essentially 0 (Fig. 5c).

Our study has demonstrated the feasibility of using HS-SPME/GC-MS to profile the VOCs in rice grains. In addition, we found that Wuchang rice can be unambiguously distinguished from non-Wuchang rice by eight putative biomarkers. Thus, this work represents an important contribution to the field of non-destructive geographical authentication of rice. Application of the method in industrial settings would bring a broad impact on the production and management of Wuchang rice, commercial rice trading and consumer's satisfaction. Putative biomarkers include 2AP, consistent with its contribution to rice aroma and previous findings that 2AP can serve as a biomarker to distinguish fragrant rice from non-fragrant ones. More importantly, our untargeted metabolomic approach also identified other putative biomarkers of diverse chemical properties. In the following section, we discuss the relevance of these compound in distinguishing rice verities.

Among the eight putative biomarkers, 2-AP is the most important source of flavor in rice, followed by aldols and ketones. As the most important aroma compound in fragrant rice, 2-AP promotes appetite and improves human metabolim^32–34^. The odor of 2-AP at 0.05 ppm aroma was described as popcorn, which is positively correlated with “butter” and “corn. The odor threshold for 2-AP in water and air had been determined to be 0.1 nL/L, and 0.02 ng/L, respectively^35^. The extremely low threshold makes 2-AP an important source of food aroma. In rice, 2-AP is mostly produced by the plants, although it was originally thought as a product of the Maillard reaction during rice cooking^36,37^. In line with this, we detected 2-AP by GCMS in the rice grain with the highest concentration observed in the Wuchang samples. These results are also in consistent with previous reports that different levels of 2-AP are detected in distinct rice varieties^38,39^. For instance, a 10 times lower level of 2-AP was found in non-fragrant rice compared to that of fragrant rice. Together with other compounds such as hexanal, octanal, (E,E)-2,4-decanedialdehyde, (E)-2-nonanedialdehyde, 4-vinyl-2-methoxy -phenol and hydrazine, 2-AP has been identified as a common aromatic compound among three fragrant rice varieties^40^. Thus, the high level of 2-AP in Wuchang rice suggests that 2-AP is a major factor in determining the pleasant flavor of Wuchang rice.

Aldehydes were odorous compounds found in many plants and foods, especially in fragrant rice. They are generated either by the oxidation of free fatty acids or the decomposition of linoleic acid^41^. We found three aldehydes that show differential levels between Wuchang and non-Wuchang rice: heptanal, octanal, and (E)-2-decenal. Previous studies have shown that heptanal, among other saturated aldehydes such as valeraldehyde, hexanal, heptaldehyde, octanal, and furfural, are odor-generating compounds in rice. Interestingly, heptanal and octanal give rice a pleasant fresh grassy scent and a light fruit scent at low concentration, while it produces a disgusting rancid taste if the concentration is too high. In addition, (E)-2-nonenal have a pleasant orange aroma at an extremely low concentration. It will be of interest to explore the contribution of these compounds, either alone or combined with other compounds, in rice flavor.

Three alcohols, 1-heptanol, (E)-2-decen-1-ol, 3,7,11-Trimethyl-3-dodecanol, were identified in our samples. These volatile alcohols produce a milder odor, e.g., the turf taste of 1-heptanol. Most alcohol compounds have higher thresholds, and only higher contents or a non-saturated alcohol such as (E)-2-nonenol and 3,7,11-trimethyl-3-dodecyl alcohol will have a great influence on the flavor.

Ketones can be generated from a variety of pathways in plants, including oxidative or thermal degradation of polyunsaturated fatty acids, amino acid degradation or microbial oxidation^42^. Ketones give rice a pleasant aroma. Daygon et al. reported that compounds such as 2-heptanone, 2-hexanone, and 3-octene-2-one give fruity/floral aroma in rice, contributing to rice flavor^43^. We observed a narrow distribution of 3-octene-2-one content in Wuchang rice (Fig. 3a), indicating a potential of 3-octene-2-one in determining the geographic origin of rice. Interestingly, 3-octene-2-one is considered to be the most active ketone in rice, with a rose aroma and excellent long-lasting flavor, leading to the most intense flavor of rice.

## Conclusion

A total of 22 volatile compounds were identified by HS–SPME–GC–MS. Among the identified compounds, aldehydes, alcohols, ketones, hydrocarbon were observed to be predominance coumpounds. Among them, heptanal, octanal, (E)-2-decenal, 1-heptanol, (E)-2-decen-1-ol, 3,7,11-Trimethyl-3-dodecanol, 3-octene-2-one, and 2-acetyl-1-pyrroline was used to differentiate Wuchang rice from the others using PCA analysis. The amount of 2-acetyl-1-pyrroline was significant higher than the others. 2-AP together with the other 7 compouds constitute the flavor characteristics of Wuchang rice. The methods can be considered to analyze the rice flavor trait of different cultivars.

## Supplementary Information

Supplementary Tables.

Supplementary Table S3.

## Data Availability

The data that support the findings of this study are available from the corresponding author L M upon reasonable request.
