# Supplementary material for: Analysis of volatile compounds by GCMS reveals their rice cultivars
Source: Sci Rep. 2023 May 17;13:7973. doi: 10.1038/s41598-023-34797-2 (PMC10192344; doi:10.1038/s41598-023-34797-2)
Supplement: Supplementary file 1 — Supplementary Tables. [file 41598_2023_34797_MOESM1_ESM.doc]

Table s1 Information of the 22 volatile compounds. (RT indicates the retention time of each compound,

RI and RI^a^ represent the experimental retention index and the reference retention index，RI^a^ values were obtained from https://www.nist.gov/)

| Name | RT | RI | RI^a^ | Molecular formula |
| --- | --- | --- | --- | --- |
| **Aldehyde** |  |  |  |  |
| Hexanal | 10.63 | 848 | 856 | C_6_H_12_O |
| Heptanal | 14.06 | 839 | 844 | C_7_H_14_O |
| (Z)-2-Heptenal | 16.09 | 874 | 899 | C_7_H_12_O |
| Octanal | 17.98 | 907 | 941 | C_8_H_16_O |
| (E)-2-Octenal | 20.13 | 763 | 792 | C_8_H_14_O |
| (E)-2-Nonenal | 21.79 | 697 | 777 | C_9_H_16_O |
| Decanal | 24.17 | 839 | 877 | C_10_H_20_O |
| (E)-2-Decenal | 25.42 | 804 | 856 | C_10_H_18_O |
| **Alcohol** |  |  |  |  |
| 1-Heptanol | 16.53 | 739 | 754 | C_7_H_16_O |
| 1-Octen-3-ol | 16.96 | 841 | 892 | C_8_H_16_O |
| (E)-2-Decen-1-ol | 20.38 | 778 | 790 | C_10_H_20_O |
| 2-butyl-Octanol | 22.53 | 733 | 798 | C_12_H_26_O |
| 3,7,11-trimethyl-3-Dodecanol | 25.82 | 780 | 817 | C_15_H_32_O |
| **Ketones** |  |  |  |  |
| 3-Octen-2-one | 19.35 | 800 | 810 | C_8_H_14_O |
| Isoshyobunone | 28.80 | 733 | 761 | C_15_H_24_O |
| **Heterocyclic compound** |  |  |  |  |
| 2-Acetyl-1-pyrroline | 14.76 | 897 | 901 | C_6_H_9_NO |
| 2-pentyl-Furan | 17.37 | 864 | 885 | C_10_H_20_O |
| **Hydrocarbon** |  |  |  |  |
| D-Limonene | 19.13 | 872 | 884 | C_10_H_16_ |
| 2,3,5,8-tetramethyl-Decane | 19.88 | 754 | 796 | C_14_H_30_ |
| Dodecane | 23.98 | 822 | 848 | C_12_H_26_ |
| 2,6,10,15-tetramethyl-  Heptadecane | 25.52 | 830 | 852 | C_21_H_44_ |
| Heptacosane | 27.21 | 747 | 766 | C_27_H_56_ |

Table S2 Siloxane derivatives detected using SPME GC/MS. (RT indicates the retention time of the compound, RI and RI^a^ represent the experimental retention index and the reference retention index, respectively. RI^a^ values were obtained from https://www.nist.gov/)

| Number | Name | RT | RI | RI^a^ | Molecular formula |
| --- | --- | --- | --- | --- | --- |
| 1 | Cyclotrisiloxane, hexamethyl- | 11.19 | 859 | 885 | C_6_H_18_O_3_Si_3_ |
| 2 | Silane, trimethyl[[1-[(trimethylsilyl)ethynyl]cyclohexyl]oxy]- | 23.35 | 779 | 724 | C_14_H_28_OSi_2_ |
| 3 | 2',6'-Dihydroxyacetophenone, bis(trimethylsilyl) ether | 28.46 | 709 | 713 | C_14_H_24_O_3_Si_2_ |
| 4 | 1-Monolinoleoylglycerol trimethylsilyl ether | 32.94 | 608 | 617 | C_27_H_54_O_4_Si_2_ |
| 5 | Pentasiloxane, dodecamethyl- | 33.89 | 690 | 692 | C_12_H_36_O_4_Si_5_ |
| 6 | Hexasiloxane, tetradecamethyl- | 35.59 | 696 | 727 | C_14_H_42_O_5_Si_6_ |
| 7 | Heptasiloxane, hexadecamethyl- | 36.96 | 647 | 658 | C_16_H_48_O_6_Si_7_ |
